# Chemically modified microRNA delivery via DNA tetrahedral frameworks for dental pulp regeneration

**DOI:** 10.1186/s12951-024-02393-9

**Published:** 2024-04-04

**Authors:** Xiaoling Wei, Huaxing Xu, Mengqi Zhou, Qiangqiang Zhou, Mingqiang Li, Yuehua Liu

**Affiliations:** 1 Shanghai Stomatological Hospital and School of Stomatology, Fudan University, Shanghai, 200001 China; 2 Shanghai Key Laboratory of Craniomaxillofacial Development and Diseases, Fudan University, Shanghai, 200001 China; 3 School of Chemistry and Chemical Engineering, New Cornerstone Science Laboratory, Frontiers Science Center for Transformative Molecules, National Center for Translational Medicine, Shanghai Jiao Tong University, Shanghai, 200240 China

**Keywords:** Dental pulp regeneration, Dental pulp stem cells, Angiogenesis, Nucleic acid modifications, Tetrahedral-framework nucleic acid nanostructure

## Abstract

Dental pulp regeneration is a promising strategy for addressing tooth disorders. Incorporating this strategy involves the fundamental challenge of establishing functional vascular networks using dental pulp stem cells (DPSCs) to support tissue regeneration. Current therapeutic approaches lack efficient and stable methods for activating DPSCs. In the study, we used a chemically modified microRNA (miRNA)-loaded tetrahedral-framework nucleic acid nanostructure to promote DPSC-mediated angiogenesis and dental pulp regeneration. Incorporating chemically modified miR-126-3p into tetrahedral DNA nanostructures (miR@TDNs) represents a notable advancement in the stability and efficacy of miRNA delivery into DPSCs. These nanostructures enhanced DPSC proliferation, migration, and upregulated angiogenesis-related genes, enhancing their paracrine signaling effects on endothelial cells. This enhanced effect was substantiated by improvements in endothelial cell tube formation, migration, and gene expression. Moreover, in vivo investigations employing matrigel plug assays and ectopic dental pulp transplantation confirmed the potential of miR@TDNs in promoting angiogenesis and facilitating dental pulp regeneration. Our findings demonstrated the potential of chemically modified miRNA-loaded nucleic acid nanostructures in enhancing DPSC-mediated angiogenesis and supporting dental pulp regeneration. These results highlighted the promising role of chemically modified nucleic acid-based delivery systems as therapeutic agents in regenerative dentistry and tissue engineering.

**Graphical abstract:**

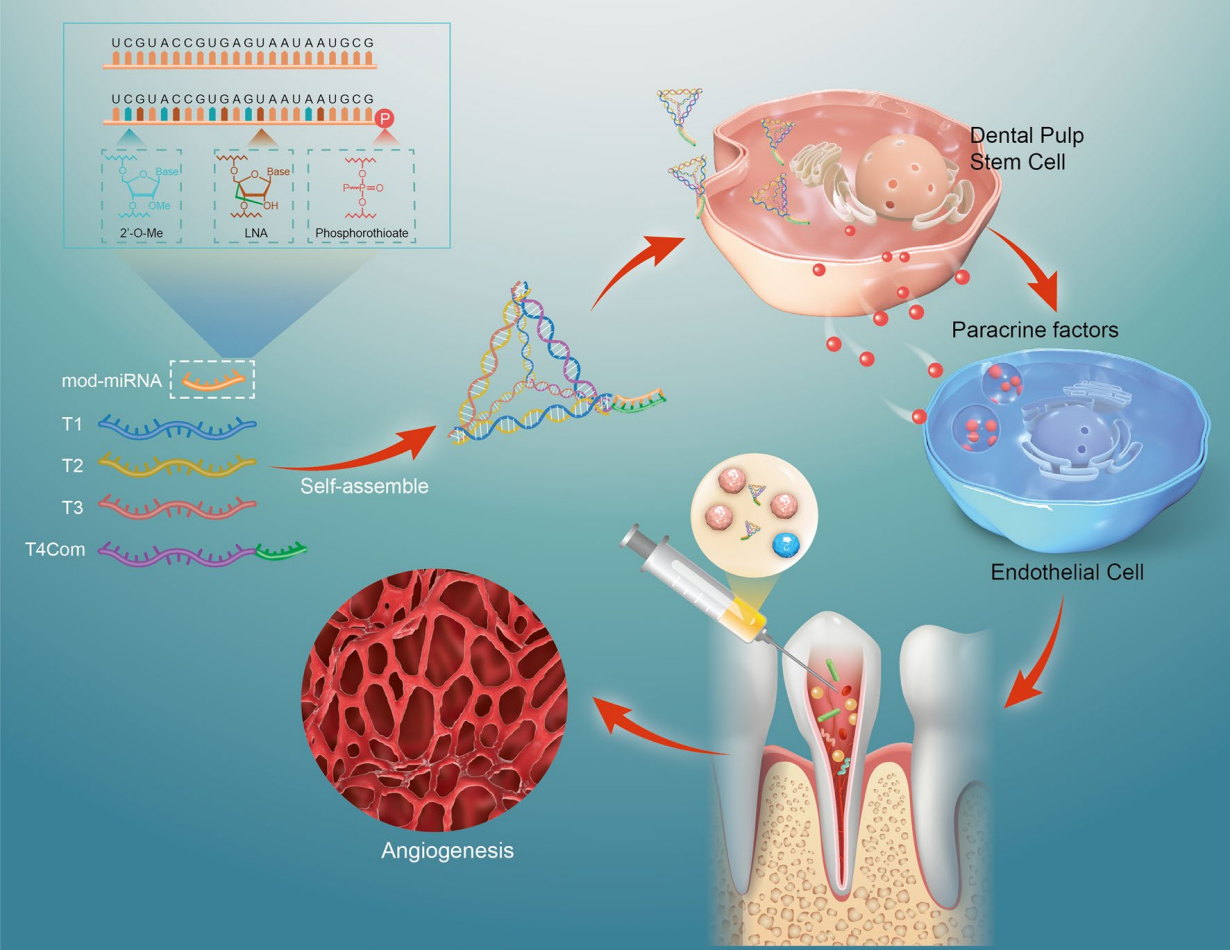

## Introduction

Dental pulp is an indispensable oral tissue and plays a crucial role in supplying nutrients, forming dentin, performing sensory functions, and facilitating defensive reactions. Conditions such as deep caries, tooth trauma, and severe periodontitis often lead to pulpitis, necessitating traditional root canal therapy (RCT) and potentially resulting in tooth discoloration and increased tooth fragility [1].

In 2005, researchers made a significant breakthrough when exploring the regeneration of the dentin-pulp complex through stem cell transplantation.They uncovered the potential of dental pulp stem cells (DPSCs) for pulp regeneration [2]. These cells, strategically located within the dental pulp microenvironment close to blood vessels, play a pivotal role in influencing angiogenesis and the formation of new blood vessels [3]. They exhibit remarkable paracrine abilities and release growth factors and cytokines essential for advancing angiogenesis. This secretion orchestrates the proliferation and migration of endothelial cells and the assembly of capillary-like structures. Furthermore, DPSCs perform paracrine signaling to regulate the expression of angiogenic factors, fostering an environment conducive to the sprouting and extension of blood vessels [4–6]. Therefore, activating DPSCs has become essential in the rapid establishment of functional vascular networks during dental pulp regeneration.

Numerous therapeutic approaches have been developed to activate the vascular potential of DPSCs [7–9]. Among these, microRNAs (miRNAs) have emerged as promising tools to drive stem cell-mediated angiogenesis. As small non-coding RNAs, miRNAs play a crucial role in orchestrating post-transcriptional gene expression and intricately influence various cellular processes, including angiogenesis. Leveraging the angiogenic processes of miRNAs can positively influence vascular network formation and dental pulp regeneration [10, 11]. Despite their potential for regulating cellular processes, miRNAs have certain inherent shortcomings. They are susceptible to degradation by ribonucleases, which are present in bodily fluids and tissues [12]. Furthermore, the efficient delivery of miRNAs to target cells, including DPSCs, is hindered by challenges such as size, charge, and structural barriers, ultimately leading to a low delivery efficiency [13]. Therefore, it is crucial to address the issues associated with miRNA delivery and stability to fully harness their therapeutic potential for facilitating dental pulp regeneration.

Currently, researchers are actively exploring innovative delivery strategies, which include using lipid-based nanoparticles, viral vectors, and modified RNA molecules, to enhance miRNA stability and improve its cellular uptake to induce potent angiogenic effects [14]. Chemical modifications have also been shown to increase the stability and half-life of a miRNA and protect it from enzymatic degradation [15]. Moreover, novel drug delivery systems, such as DNA nucleic acid frameworks, can potentially revolutionize delivery efficiency by acting as controlled-release scaffolds for miRNAs [16]. These frameworks offer spatial and temporal control over miRNA delivery, enabling the precise targeting of cells and facilitating cellular uptake [17]. However, it remains uncertain whether tetrahedral DNA nanostructure (TDN) delivery systems can effectively and reliably transport miRNAs to enhance dental pulp regeneration.

Herein, we present a chemically modified tetrahedral-framework nucleic acid (TDN)-based delivery system for microRNAs (miRNAs) that enhances DPSC-mediated angiogenesis and dental pulp regeneration. This strategy not only enhances miRNA transduction, but also improves the activities and efficacies of miRNAs that promote the vascular potential of DPSCs. Our research provides novel insights into dental pulp regeneration, paving the way for revolutionary advancements in this field.

## Methods

### Chemical modification of miR-126-3p

The rationale for selecting specific positions for chemical modifications in our study is based on their functional importance in enhancing the stability, specificity, and efficacy of the miRNA molecule. 2’-O-methyl modification is widely used and can enhance the binding of the drug to the target mRNA, inhibit nucleases’ hydrolysis, reduce in vivo immunogenicity, and impart some lipophilicity to the nucleic acid structure. Similarly, 2’-fluoro modification can enhance miRNA’s affinity and stability. In our study, we chose to introduce 2’-O-methyl modifications and 2’-fluoro modifications at internal positions to improve stability and resistance to nucleases, thereby enhancing miRNA efficacy and bioavailability. Additionally, Phosphorothioate modifications at the 3’ and/or 5’ ends were employed to protect the miRNA from exonucleases, increasing its stability. Locked nucleic acid (LNA) is a conformationally restricted modification that adopts a C3’-endo conformation, maintaining high target affinity and nuclease resistance with a shorter sequence [18]. We incorporated LNA modifications at internal positions to enhance binding affinity and specificity towards target mRNAs, thus improving the miRNA’s effectiveness in target mRNA regulation.

**Fig. 1:**
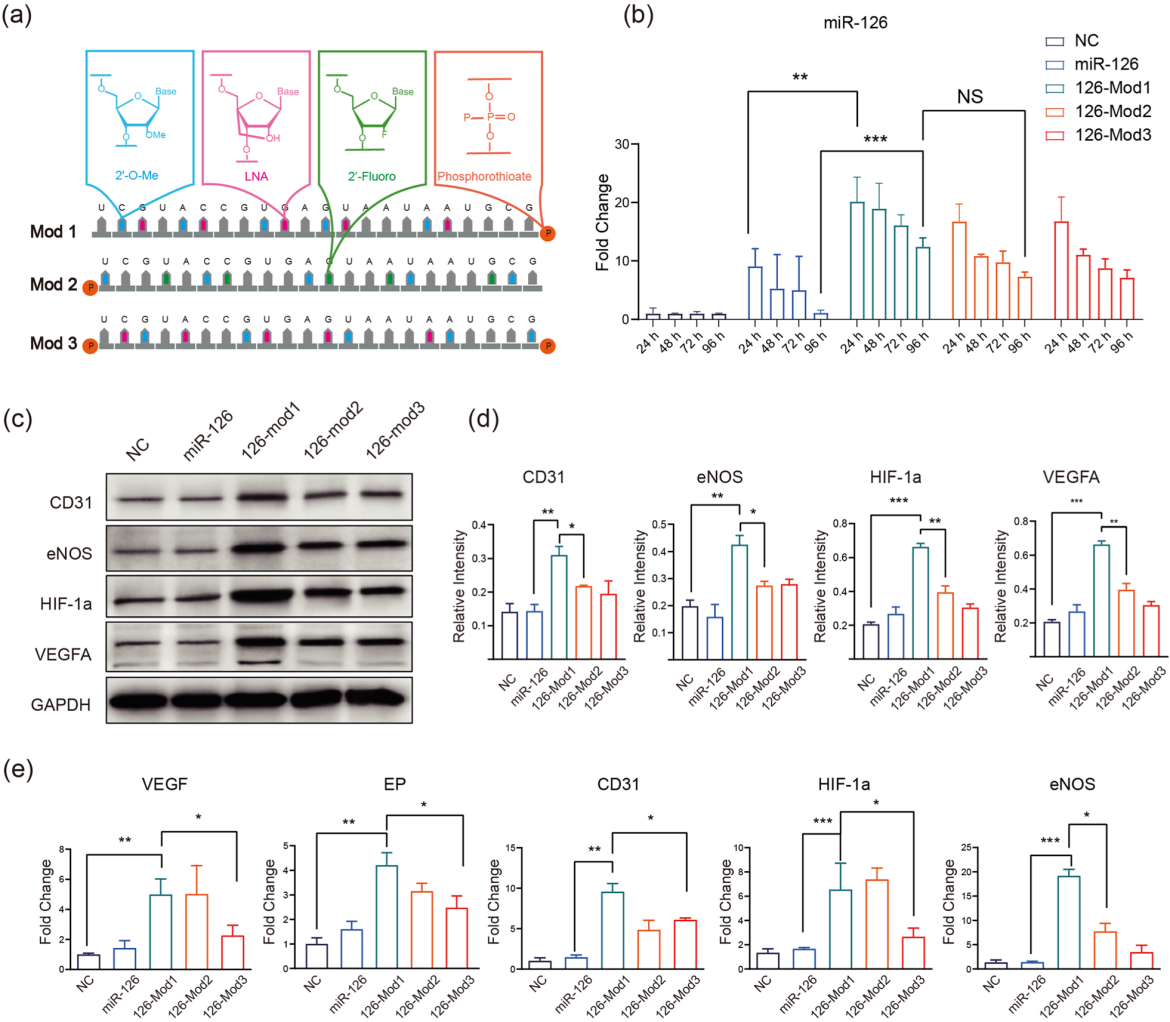
Chemical modifications of microRNAs and functional validation. **a** Design schematic of three chemical modification approaches for miR-126-3p. **b** Stability validation of miR-126-3p after three different chemical modifications: The unmodified miR-126-3p (miR-126) and the modified versions (126-mod1, 126-mod2, 126-mod3) were transfected into DPSCs (Dental Pulp Stem Cells), and the level of miR-126 is assessed at different time points (24, 48, 72, 96 h) using qPCR. **c** Functional validation of chemically modified microRNAs: WB analysis of the expression of angiogenesis-related proteins after transfection (at 96 h post-transfection) of different versions of miR-126 into DPSCs. **d** The presentation of related proteins in (**c**) was quantitatively analyzed. **e** qPCR analysis of the expression of angiogenesis-related genes after transfection of different versions of miR-126 into DPSCs. Data include the mean ± SD, statistical analysis: $$\hbox {n}=3$$, $$*p<0.05, **p<0.01, ***p<0.001$$, NS, no significance

The sequence of hsa-miR-126-3p (UCGUACCGUGAGUAAUAAUGCG) and three chemical modification schemes are shown in Fig. 1a. In modification method 1 (126-Mod1), we introduced 2’-O-methyl modifications at positions 2, 5, 9, 12, and 17. Additionally, we fortified the structure of the miRNA by adding a protective phosphorothioate modification at the 3’ end. Furthermore, we incorporated LNA modifications at positions 3, 6, 10, 13, and 18, enhancing binding affinity and specificity. Regarding modification method 2 (126-Mod2), we introduced 2’-O-methyl modifications at positions 1, 6, 11, 16 and 21. Then, we added a phosphorothioate modification at the 5’ end to safeguard it. Moreover, at positions 4, 7, 11, 14, and 19, we harnessed the power of 2’-fluoro modifications, fortifying miRNA resistance against the degradative forces of nucleases. Concerning modification method 3 (126-Mod3), we orchestrated 2’-O-methyl modifications at positions 3, 8, 13, 18, and 22, contributing to the overall structural robustness. To further improve the stability, we introduced phosphorothioate modifications at both the 3’ and 5’ ends. Furthermore, we introduced LNA modifications at positions 2, 5, 9, 12, and 17, harnessing their unique affinity-enhancing properties to refine the target interactions.

### Fabrication and characterization of miR@TDNs

**Fig. 2:**
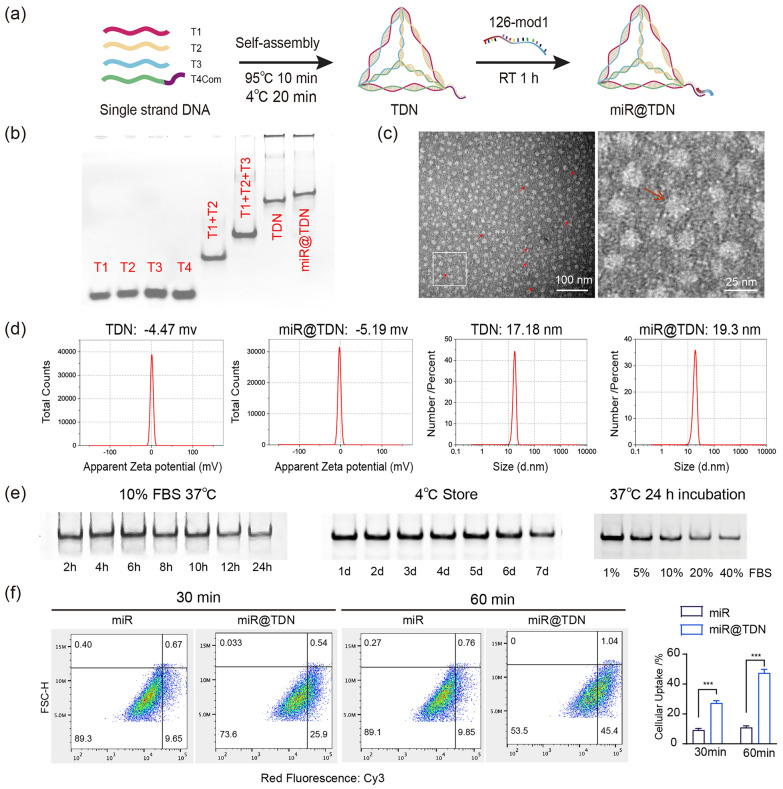
Preparation and characteristics of the miR@TDNs. **a** Schematic diagram of miR@TDN fabrication. **b** Verification of the successful synthesis of miR@TDNs using 8% PAGE. **c** Obtained TEM images of the molecular structure of miR@TDNs, the red arrow in the figure indicates the typical tetrahedral shape of miR@TDN. **d** Average zeta potentials and average sizes of the TDNs and miR@TDNs detected by DLS ($$\hbox {n}=3$$). **e** Structure stability of miR@TDN (left: Serum stability of miR@TDNs within 24 h in 10% FBS; Middle: Storage stability of miR@TDN at $$4^{\circ }\hbox {C}$$; Right: Stability of miR@TDNs in different concentrations of FBS). **f** The uptake of miR@TDN by DPSC after 30 mins and 60mins by Flow cytometer analysis, and quantitative statistics of cellular uptake from flow cytometer detection ($$\hbox {n}=3$$). Data include the mean ± SD, statistical analysis: $$***p<0.001$$

The complementary pairing of the designed nucleic acids (T1, T2, T3, T4Com, and miR-126-3p) resulted in the creation of miR@TDNs, as illustrated in Fig. 2a; nucleic acid sequences cited in previous studies are detailed in Additional file 1: Table S1 in the Supporting Information [19]. The sequences were procured from Shenggong Bioengineering Co., Ltd. (Shanghai, China). Specifically, the 3’ end of T4Com was linked to the miR-126-3p sequence, with the 3’ end of miR126-3p attached to Cy3-labeled fluorescent markers. To synthesize miR@TDNs, four individual single-stranded DNAs were combined in equal proportions in a TM buffer. This buffer contained 50 mmol/L of $\hbox {MgCl}_2 \cdot 6\hbox {H}_{2}\hbox {O}$ and 10 mmol/L of Tris-HCl. The mixture was rapidly heated to a temperature of $95\,^{\circ }\hbox {C}$ and held at this temperature for 10 min. Following this, it was cooled to $4\,^{\circ }\hbox {C}$ over 20 min. Subsequently, miR-126-3p mimics with complementary sticky ends were integrated into the synthesized miR@TDNs. Subsequently, thorough mixing and vibration were performed to ensure proper integration, after which the solution was incubated at $20\, ^{\circ }\hbox {C}$ for 2 h. Any impurities were removed by ultrafiltration. Base-pair analyses of ssDNA, TDNs, and miR@TDNs were conducted using 8% sodium dodecyl sulfate-polyacrylamide gel electrophoresis (SDS-PAGE). Transmission electron microscopy (TEM) was utilized to investigate the morphology of miR@TDNs, while a dynamic light scattering (DLS) instrument (Zetasizer Nano ZS90, Malvern) was employed to measure the $\zeta$ potential and size of both bare TDNs and miR@TDNs.

### Cell culture

DPSCs were extracted from the healthy third molars of human subjects aged between 18 and 22 years, following previously established methods [20]. All procedures involving human subjects were conducted in accordance with the ethical standards of the Ethics Committee of Shanghai Stomatological Hospital (Approval No. [2023]002) and with the 1964 Helsinki declaration and its later amendments or comparable ethical standards. Informed consent was obtained from all individual participants included in the study. Isolated cells were cultured in alpha-minimum essential medium ($\alpha$-MEM) supplemented with 20% fetal bovine serum (FBS). Human umbilical vein endothelial cells (HUVECs) were commercially obtained from ATCC. Both types of cells were incubated at $37\,^{\circ }\hbox {C}$ in an atmosphere containing 5% CO2.

### Tube formation assay of HUVECs

The tube formation assay was conducted according to a previously established protocol [21]. In summary, 2 × 10^4^ HUVECs in 50µL of endothelial medium with 10% bovine serum were seeded onto a matrigel-coated tube formation slide (Ibidi). Images were captured 4 h after seeding, and the results were analyzed using the ImageJ software.

### Detection of the migration capacities of the DPSCs

The migratory potential of the DPSCs was determined using both wound-healing and transwell experiments. In advance of each experiment, DPSCs were subjected to a 6-h serum-free medium starvation period. In the wound-healing assay, a 1000-µL pipette tip was used to create a cell-free area in 6-well plates after the formation of monolayer cell sheets. Subsequently, DPSCs were cultured with either 1% FBS/ α-MEM or supplemented with phosphate buffered saline (PBS), 200 nM of miR, 200 nM of TDNs, or 200 nM of miR@TDNs for 24 h. The µm transwell assay was performed using inserts with an 8.0- pore size in 24-well plates (Corning, USA). Specifically, 5 × 10^4^ DPSCs in 200µL α-MEM were placed in the upper chambers, while 500µL of the aforementioned medium was added to the lower chambers. After 24 h of incubation, the DPSCs in the upper chamber were removed using a swab, and the cells that had invaded and migrated were stained with crystal violet and quantified using the ImageJ software.

### In vivo matrigel plug assay

All the experimental procedures that had been conducted adhered to ethical guidelines and were approved by the Ethics Committee of Shanghai Stomatological Hospital (Approval No. [2023]002). The matrigel plug assay protocol, previously detailed, involves injecting a mixture of cells and matrigel into the subcutaneous space, which solidifies upon reaching body temperature [22]. Over time, newly formed blood vessels infiltrate the plug. The experiment consisted of six randomly assigned groups, each containing three BALB/c nude mice aged 6–8 weeks and weighing 20–23 g. A 300-$\mu \hbox {L}$ matrigel mixture, which included $100 \mu \hbox {L}$ of $1\times 10^6$ HUVECs, was subcutaneously injected into the dorsum area of each mouse. After 14 days, the mice were euthanized, and the implanted matrigel plugs were extracted from the subcutaneous tissues of the mice.

### Ectopic pulp regeneration in a nude mouse model

A nude mouse model was established following the methodology outlined in previous research [23]. Forty healthy premolars were extracted from patients undergoing orthodontic therapy, with informed consent obtained. All extracted teeth were healthy and intact. The teeth were segmented into root segments (RSs) measuring 6–8 mm in length using fissure burs. These RSs were cleaned with sterile PBS via ultrasonication for 10 min, a procedure repeated thrice. The RSs were then exposed to 17%, 10%, and 5% ethylenediaminetetraacetic acid (EDTA) in PBS for 10 min and immersed in a 1% penicillin/streptomycin solution at $4\,^{\circ }\hbox {C}$ for over three days. The experimental groups were designed as follows: the PBS group (untreated DPSC + HUVECs), the TDN group (TDN-treated DPSCs + HUVECs), and the miR@TDN group (miR@TDNs-treated DPSCs + HUVECs). Each group consisted of DPSCs and HUVECs in a 3:1 ratio. The DPSC-HUVEC mixture was encapsulated in a Type I collagen hydrogel (Nitta Gelatin, Osaka, Japan), forming a cell-laden gel composite. Each experimental group included 6 samples. After a 48-h in vitro culture period, these cell-laden hydrogel composites were implanted into the dissected dental pulp cavities of the nude mice (BALB/c nude mice aged 6–8 weeks and weighing 20–23 g). Subcutaneous implantation provided a controlled environment for monitoring the subsequent dental pulp regeneration process. After 6 weeks, the transplants were carefully harvested and fixed in 4% paraformaldehyde for 8 h. They then underwent an eight-week demineralization process using 17% EDTA at $37\, ^{\circ }\hbox {C}$, after which the specimens were prepared for histological analysis.

### Histology analysis

The samples were encased in paraffin blocks and sliced into sections with a thickness of $4 \mu \hbox {m}$. Hematoxylin and eosin (H & E) staining was conducted to assess the morphological features of the samples, particularly the neovascularization of the matrigel Plug and regenerated pulp-like tissues. Immunohistochemistry was performed using human-specific CD31 antibodies (Proteintech, Wuhan, China) to detect vascular endothelial cells (blood vessel linings). The areas of vessels in the H &E images and lumens lined by $\hbox {CD31}^{+}$ cells in the immunohistochemistry (IHC) images were measured using ImageJ software. To ascertain whether the regenerated pulp-like tissue had originated from the host or donor, immunofluorescence was performed using the human-specific mitochondrial antibody heat shock protein 90 (Abcam, Cambridge, UK).

### Statistics

Statistical analyses were conducted using the SPSS 22.0 software (IBM) and Prism 6.0 software (GraphPad Software). The data were presented as mean values with standard deviations (SD). Two-tailed unpaired t-tests were employed for two-group comparisons; One-way analysis of variance (ANOVA) was applied for normally distributed data in comparisons involving more than two groups, while the Kruskal-Wallis test was employed for samples that were either non-normally distributed or of small size. *p* <0.05 indicated statistical significance. The experiments were independently repeated at least three times, and each experiment included triplicate samples to ensure reproducibility.

## Results

### Chemical modifications enhance miR-126-3p stability and angiogenic potential

Four microRNAs (miR-126-3p, miR-21-3p, let-7b-5p, and miR-210-3p) were selected due to their established roles in promoting angiogenesis. Among these microRNAs, miR-126-3p exhibited the highest stability and strongest angiogenic potential, as illustrated in Additional file 1: Figs. S1 and S2. This section aims to explore three distinct chemical modification strategies (depicted in Fig. 1a) for enhancing the stability and functionality of miR-126-3p. We employed 2’-O-methylribonucleotide (2’-O-methyl), 2’-Fluoro-deoxyribonucleotide (2’-Fluoro), phosphorothioate backbone, and locked nucleic acid (LNA) to modify various positions within the RNA chain (Fig. 1a), with the objective of fortifying the stability of miR-126-3p to ensure its sustained presence and efficacy within cells. Upon implementing these chemical modification strategies, we thoroughly evaluated the stability of the three modified miR-126-3p variants. Transfection experiments were conducted in DPSCs, and the levels of miR-126 was monitored using qPCR at 24, 48, 72, and 96 h post-transfection. The results revealed significantly elevated levels of miR-126 variants (126-Mod1, 126-Mod2, 126-Mod3) at 24h, 48h, 72h, and 96h post-transfection in DPSCs compared to unmodified miR-126 (Fig. 1b). This suggests that all three modified miR-126-3p variants exhibited notably enhanced stability relative to the unmodified miR-126 counterpart. After confirming the stability of our modified miR-126-3p variants, we proceeded to investigate whether the angiogenic potential of miR-126 was impacted by the chemical modifications. Subsequently, supernatants were collected from DPSCs transfected with the three chemically modified miR-126 variants (at 96 h post-transfection), and qPCR and Western blot experiments were performed to analyze the expression of relevant angiogenic markers. Protein analysis revealed significantly higher levels of CD31, eNOS, HIF-1$\alpha$, and VEGFA expression in the 126-Mod1 group. Compared to controls and other modification groups, 126-Mod1 notably influenced the expression of angiogenesis-related proteins in DPSCs (Fig. 1c, d). The qPCR results were consistent with the protein analysis (Fig. 1e). Based on the observed outcomes, indicating the highest stability and a significant increase in angiogenesis marker-related proteins/mRNAs, 126-Mod1 was subsequently selected for constructing the nanoscale nucleic acid framework in subsequent experiments.

### Synthesis and characterization of miR@TDNs

The synthesis of the miR@TDNs (Fig. 2a) involves the conjugation of the cohesive termini of the TDNs and the chemically modified cohesive termini of 126-Mod1 through complementary base pairing [24]. To verify the successful synthesis of miR@TDNs, we employed 8% polyacrylamide gel electrophoresis (PAGE) to examine the molecular weight distinctions among T1, T2, T3, T4, TDNs, and miR@TDNs (Fig. 2b). Additionally, by employing TEM and nano-potentiometer detection, we determined the geometric properties of miR@TDNs, unveiling their unique negatively-charged (− 5.19 ± 2.23 mV) triangular structure (Fig. 2c), which measured approximately 19.32 ± 2.799 nm. Notably, this size closely corresponded to the dimensions of TDNs (17.18 ± 3.364 nm) (Fig. 2d). To comprehensively characterize the synthesized miR@TDNs, we validated their stabilities.The miR@TDNs were stable over 24 h in 10% FBS at elevated serum concentrations, representing an optimal environment for in vitro cell viability (Fig. 2e, left). Moreover, the miR@TDNs exhibited storage compatibility at $4^{\circ }\hbox {C}$ for up to 7 days (Fig. 2e, middle). Furthermore, the miR@TDNs remained stable for 24 h in 40% FBS, consistent with the results from previous research (Fig. 2e, right) [25]. Next, we employed flow cytometry and immunofluorescence to investigate the cellular uptake of miR@TDNs by DPSCs. The fluorescence microscopy results revealed that the DPSCs had exhibited a consistent and uniform cytoplasmic distribution of miR@TDNs, whereas the unlinked miRNAs (miRs) had displayed a significantly lower uptake (Fig. S3). The flow cytometry analysis revealed significant differences in the cellular uptake efficiencies of the miR@TDNs and unlinked miRs within the DPSCs; after 30 min, the DPSCs had demonstrated an uptake efficiency of 25.9% for miR@TDNs, significantly outperforming the unlinked miRs, which had demonstrated an uptake efficiency of 9.65%. After 60 min, the uptake efficiency of miR@TDNs surged to 45.4%, starkly contrasting with the static 9.85% uptake observed for the unlinked miRs (Fig. 2f).

### miR@TDNs-promoted proliferation, migration, and angiogenesis marker expression of DPSCs

**Fig. 3:**
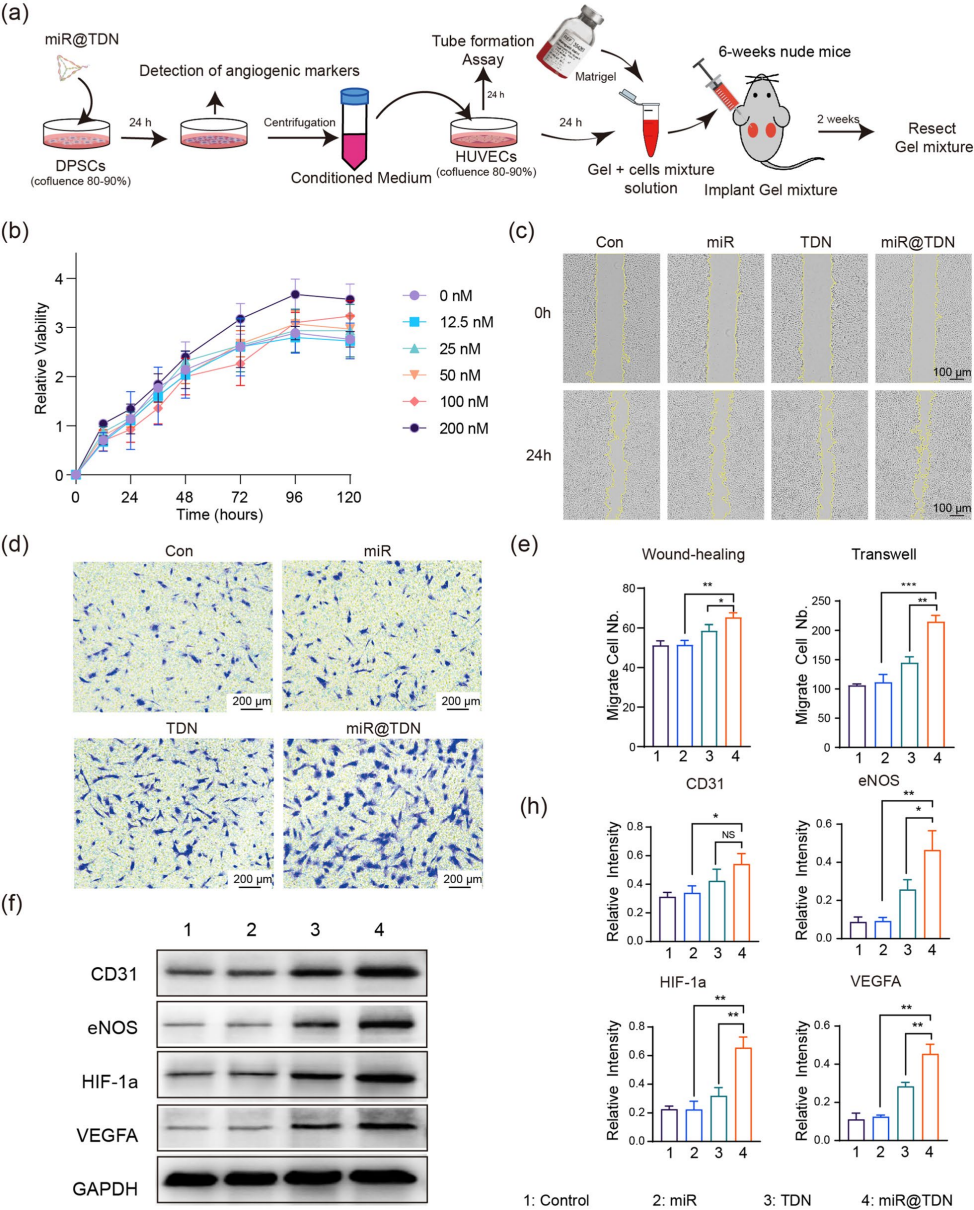
Modulation of proliferation, migration, and angiogenic marker expression of DPSCs using miR@TDNs. **a** Schematic diagram of the experimental design for miR@TDN-based modulation of the behavior of DPSCs. **b** CCK-8 assay for evaluating the cell viabilities of the DPSCs after intervention with different concentrations of miR@TDN, $$\hbox {n}=3$$. **c**, **d** Results of applying the wound-healing and transwell assays to assess the migration abilities of the DPSCs. **e** Quantification of the relative invasion and migration cell number in (**c**), (**d**), $$\hbox {n}=3$$. **f** Results of the WB analyses of the expression of vascular-related genes in DPSCs following miR, TDN, and miR@TDN interventions, $$\hbox {n}=3$$. (**h**) The quantitative analysis of the related proteins in (**f**) is performed to determine the expression levels, $$\hbox {n}=3$$. Data include the mean ± SD, statistical analysis: $$*p<0.05, **p<0.01, ***p<0.001$$

Considering the crucial role of DPSCs in dental pulp regeneration, we comprehensively evaluated the influence of miR@TDNs on DPSCs, focusing on cell proliferation, migration, and the upregulation of angiogenesis-related genes (Fig. 3a). This evaluation was performed by subjecting DPSCs to a range of miR@TDN concentrations, spanning from 12.5 to 200 nM. Subsequently, the proliferative activities of the DPSCs were monitored for 120 h (Fig. 3b). While concentrations of 12.5, 25, 50, and 100 nM only produced marginal effects on DPSC proliferation, there was a notable increase in proliferation compared to that of the control group at the 120-h mark. Notably, 200 nM of miR@TDNs significantly enhanced DPSC proliferation compared to the control group, highlighting their potential as robust stimulators of cell growth and tissue regeneration. Cell scratch (Fig. 3c) and transwell migration (Fig. 3d) assays were performed to further investigate the impact of miR@TDNs on DPSC migration. Stem cell migration enabled them to move to areas within the body where vascular development was required [26]. The data showed a significant increase in DPSC migration toward the scratch area after 24 h. Notably, the miR@TDN-treated group at a concentration of 200 nM displayed a considerably enhanced migratory response compared to the groups treated solely with miR (200 nM) or TDN (200 nM). This outcome was reaffirmed by the results of the transwell migration assay (Fig. 3e), which further substantiated the ability of miR@TDNs to enhance the migratory ability of DPSCs.Finally, the secretion profile of angiogenic factors in DPSCs following miR@TDN intervention (200 nM) was evaluated, since these molecules had the potential to influence the behavior of surrounding cells. The results of our study revealed the direct upregulation of angiogenic-related factors in DPSCs following miR@TDN intervention. Furthermore, the qPCR analysis demonstrated a significant elevation in the expression of VEGFa, CD31, eNOS, EP, and fibroblast growth factor in DPSCs treated with miR@TDN, surpassing that in both the miR and TDN groups (Additional file 1: Fig. S4). This effect was corroborated by the WB experiments, showcasing heightened expression levels of CD31, eNOS, HIF-1$\alpha$, and VEGFA in the miR@TDN-treated group (Fig. 3f, h). The qPCR and WB analysis results collectively highlighted the potent capabilities of miR@TDNs in augmenting the expression of angiogenesis-related factors within DPSCs.

### miR@TDN-enhanced angiogenesis via the paracrine signaling of the DPSCs

**Fig. 4:**
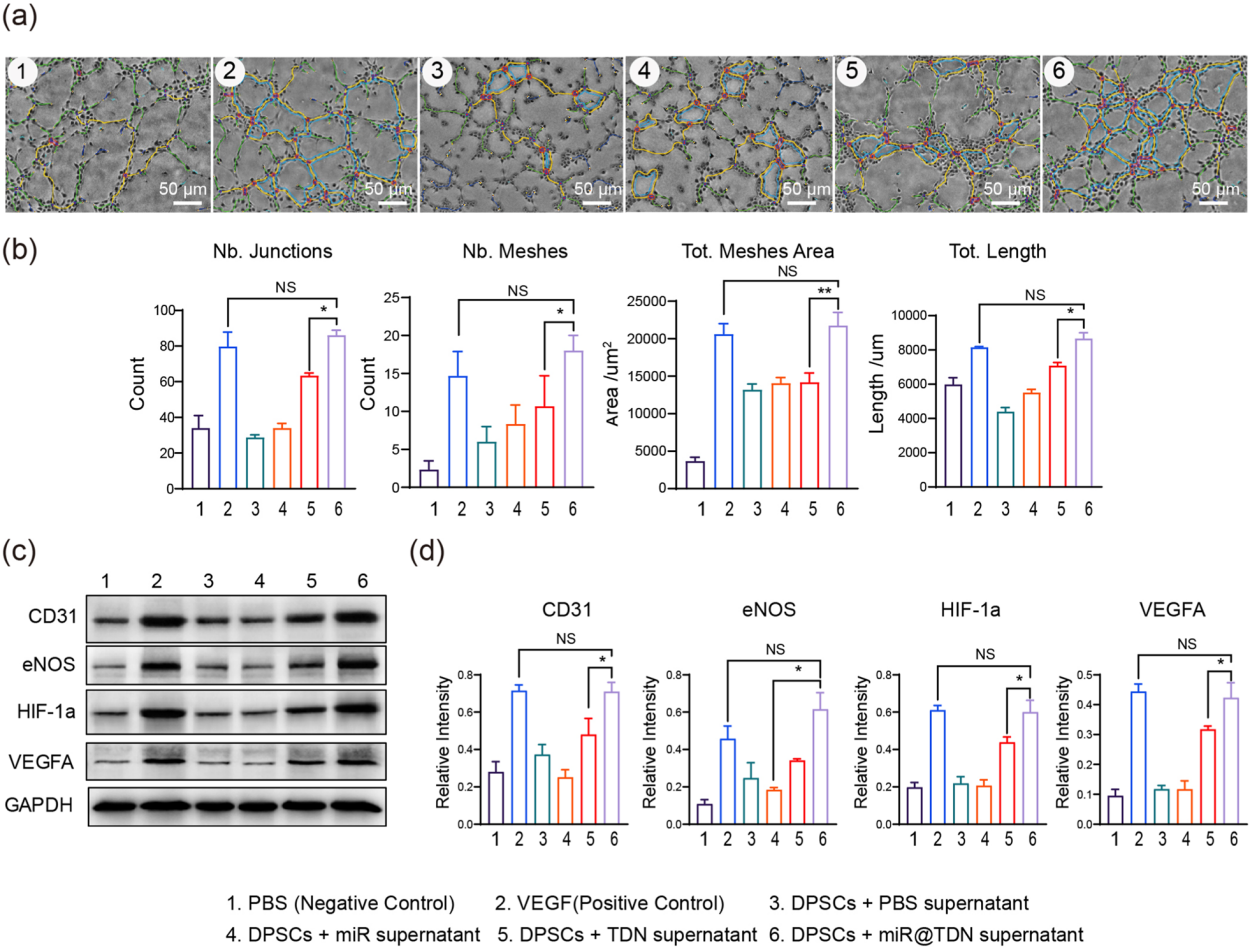
miR@TDNs- enhanced angiogenesis via the paracrine signaling of DPSCs. **a** The angiogenic potential of HUVECs is assessed through a tube formation assay: the six groups consist of supernatants from DPSCs treated with four distinct interventions (DPSCs treated with PBS, miR, TDN, or miR@TDN), along with VEGF (50 ng/ml) as a positive control and PBS as a negative control. The vascular formation status is assessed at 4 h across the different groups. **b** Quantitative analysis of the tube formation assay images from (**a**). **c** Results of WB analyses of the expression of vascular-related genes in HUVECs following six different interventions, $$\hbox {n}=3$$. **d** The quantitative analysis of the related proteins in (**c**) to assess their expression levels, $$\hbox {n}=3$$. Data include the mean ± SD, statistical analysis: $$*p<0.05, ***p<0.001$$, and NS indicates no significance

To validate the potential of miR@TDNs in triggering the initiation of paracrine signaling in DPSCs and subsequently facilitating angiogenesis in endothelial cells, we collected conditioned medium (CM) from DPSCs that had undergone various interventions (solely PBS, 200 nM miR, 200 nM TDN, 200 nM miR@TDN), as illustrated in Fig. 3a. This CM was used to stimulate HUVECs. Additionally, VEGF (50 ng/ml) served as a positive control, and PBS acted as a negative control, directly impacting HUVECs. This approach enabled the assessment of their angiogenic capabilities and angiogenesis-related marker expression [2]. These findings revealed a noteworthy enhancement in the angiogenic potential of HUVECs upon exposure to the CM from the miR@TDN-treated DPSCs. Particularly, in tube formation experiments, parameters such as Nb.Junction (the count of junctions within the structure), Nb.Mesher (the number of meshers within the structure), Tot.meshes area (the total area covered by meshes within the structure), and Tot.Length (the total length of structural elements) exhibited a substantial increase within the miR@TDN group, surpassing that in both the miR and TDN groups (Fig. 4a, b). Strikingly, this enhancement in angiogenic indicators was comparable to that observed in the VEGF group (positive control), thereby establishing the robust potential of miR@TDNs to induce angiogenesis. This equivalence was consistently supported by the WB analysis results, which revealed significantly elevated expressions of CD31, eNOS, HIF-1a, and VEGFA within the miR@TDN group, with no notable distinction from the VEGF group (Fig. 4c, d); the corresponding qPCR results also confirmed this trend (Additional file 1: Fig. S5).

**Fig. 5:**
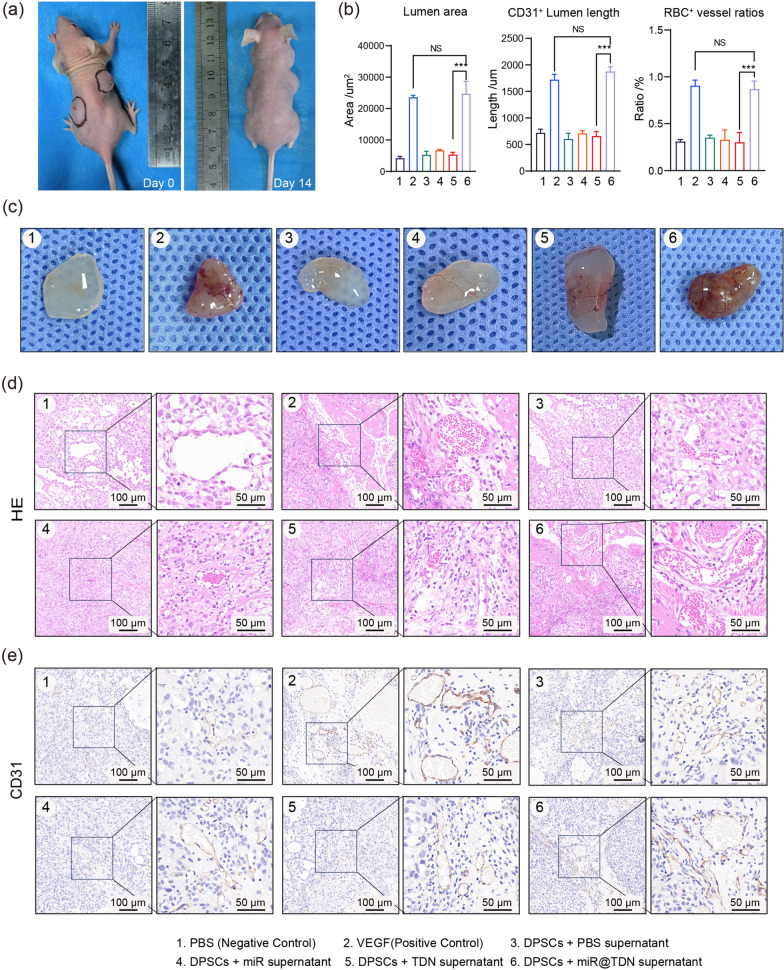
Enhancement of in vivo angiogenesis by miR@TDNs. **a** Photographs before implantation and photographs 14 days after the implantation of the gel plugs. **b** Results of statistical analysis, including the ratio of $$\hbox {RBC}^{+}$$ vessels, lumen area, and $$\hbox {CD31}^{+}$$ lumen length, $$\hbox {n}=3$$. **c** Microscopic images of matrigel plugs removed from subcutaneous tissue of nude mice 14 days post-surgery. **d** H &E staining and, **e** positive IHC staining of CD31 illustrating the formation of newly formed capillaries, $$\hbox {n}=3$$. Data presented as mean ± SD, with statistical analysis indicating $$*** p<0.001$$ and NS indicating no significance

To further validate the paracrine angiogenic effect of miR@TDNs on HUVECs, we conducted the matrigel plug assay, which is a well-established and effective method for assessing in vivo angiogenesis [27]. In this experiment, DPSCs were treated with PBS, 200 nM miR, 200 nM TDN, and 200 nM miR@TDN individually to collect their respective conditioned media (CM). The collected CM was then used to stimulate HUVECs. Concurrently, HUVECs were directly exposed to either PBS or 50 ng/ml of VEGF, serving as negative and positive controls, respectively. Subsequently, the HUVECs were embedded in matrigel and injected subcutaneously into nude mice (Fig. 5a). The the matrigel plugs were harvested two weeks postoperatively for further analysis.The procedural steps for the implantation and plug collection are illustrated in Fig. 3a.

Upon initial visual inspection, the matrigel plugs exhibited a clear distinction in terms of plug color and vessel density under the miR@TDN-treated and blank control groups (Fig. 5c). This marked difference suggested a potent angiogenic effect of miR@TDN. Furthermore, we performed H &E staining to provide direct evidence of newly formed vessels within the plugs, allowing the functional and nonfunctional lumens to be distinguished. While all the experimental groups had displayed structures resembling lumens, there were evident variations in the lumen area and the proportion of erythrocyte-containing lumens (Fig. 5d). Importantly, The quantitative analysis revealed that the miR@TDN-treated groups had exhibited a significantly higher lumen area than that of the blank control, miR-treated, and naked TDN groups while exhibiting no significant difference from that in the VEGF-treated (positive control) group (Fig. 5b). Additionally, based on the data of the $\hbox {RBC}^+$ vessel ratio (the number of $\hbox {RBC}^+$ vessels / the total number of vessel-like structures) (Fig. 5b) [28], it is evident that the miR@TDN-treated group exhibited a significantly enhanced rate of angiogenesis, characterized by many newly formed vessels containing red blood cells (RBCs) (Fig. 5d). This functional blood vessel formation indicates that the vascular system has achieved normal structural and functional characteristics, efficiently transporting oxygen, nutrients, and other vital biomolecules [29]. Immunohistochemistry (IHC) staining of CD31, a well-established endothelial marker, was performed, revealing thick $\hbox {CD31}^{+}$ lumens within the VEGF and miR@TDN groups, thereby signifying functional vessels as opposed to nonfunctional $\hbox {CD31}^{+}$ cell clusters (Fig. 5e) and reinforcing the previous findings. Adhering to these observations, our quantitative analysis demonstrated that the miR@TDN-treated groups had exhibited significantly greater $\hbox {CD31}^{+}$ lumen lengths than that of the blank control, miR-treated, and naked TDN groups, with no significant differences from that of the VEGF-treated (positive control) group (Fig. 5b).

### Transcriptome analysis of miR@TDN-induced angiogenesis

**Fig. 6:**
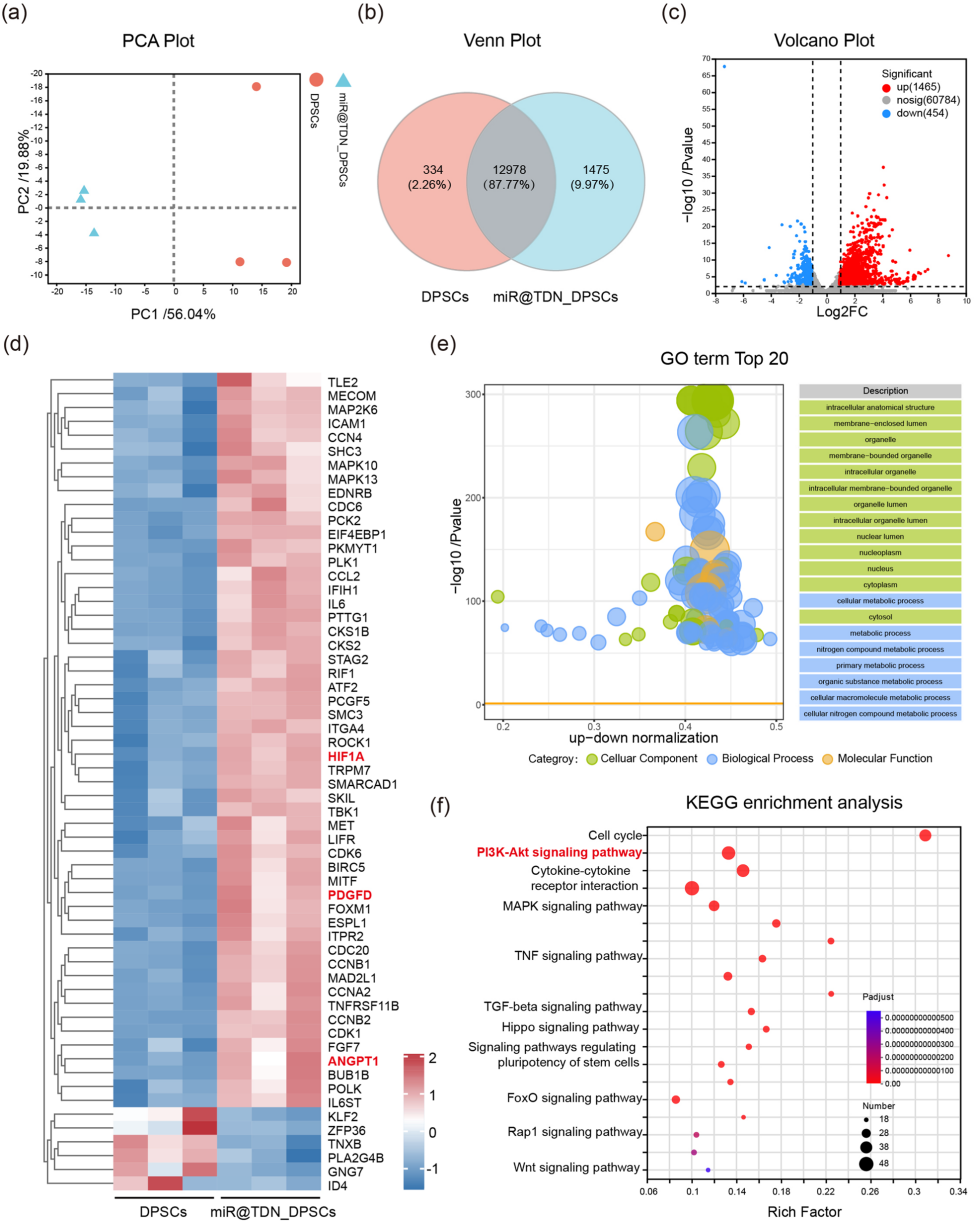
Transcriptome analysis elucidates the underlying mechanism of action in DPSCs induced by miR@TDNs. **a** Results of PCA using differentially expressed genes from the miR@TDN_DPSC and DPSC groups. Each data point represents the PCA analysis of an individual sample. **b** The Venn diagram illustrates the differentially expressed mRNAs in the miR@TDN_DPSCs and DPSCs groups. **c** The volcano plot illustrates the disparities in gene expression between the miR@TDN_DPSCs and DPSCs groups. The blue points denote the significantly reduced genes with a fold change $>2$, the red points represent the significantly upregulated genes with a fold change $>2$, and the grey points indicate the absence of any significant differences. **d** The heatmap displays the expression of genes associated with angiogenic functions in both the miR@TDN_DPSCs and DPSCs groups. **e** Results of GO enrichment analysis conducted to explore the relationship between the representative differentially expressed genes and their enriched pathways. **f** The bubble charts depict the results of the KEGG pathway analysis for differentially expressed genes (top 20 KEGG pathways of differentially expressed genes)

After verifying the abilities of the miR@TDNs in activating human DPSCs (miR@TDN-DPSCs) and the effect of their paracrine secretion in promoting dental pulp regeneration, we investigated the underlying mechanisms behind these observations using transcriptome analysis. We investigated the depth of the mRNA sequencing analyses for both DPSCs and miR@TDN-DPSCs. The principal component analysis (PCA) results illustrate the profound disparity between the gene expression profiles of miR@TDN-DPSCs and DPSCs (Fig. 6a). The Venn diagram highlights this distinction, with 12,978 genes shared between the groups and 1475 genes exclusive to miR@TDN-DPSCs (Fig. 6b). The volcano plots (Fig. 6c) emphasize 1919 DEGs, among which 1,465 were upregulated and 454 were downregulated by miR@TDN treatments (200 nM). These DEGs, which formed a subset of genes significantly altered by miR@TDNs, likely acted as drivers of the observed changes in DPSCs. The GO enrichment analysis (Fig. 6e) revealed that 20 biological processes had been predominantly affected, notably intracellular anatomical structures, membrane-enclosed lumens, and organelles. These enrichments may have profoundly affected the angiogenic potential of DPSCs. The bubble diagram visually represents the upregulation of genes associated with the “cellular components,” “biological processes,” and “molecular functions” in the miR@TDN group. Upregulated genes that play pivotal roles in angiogenesis, cell proliferation, and tissue repair are particularly interesting. The heat maps (Fig. 6d) highlight the significant upregulation of genes associated with angiogenesis and cell proliferation in the miR@TDN-DPSCs. Noteworthy genes such as HIF1$\alpha$, Platelet-Derived Growth Factor D, angiopoietin 1, and renowned pro-angiogenic factors were upregulated, suggesting that miR@TDN treatment had stimulated the production of these essential growth factors, which are crucial for cell proliferation and new blood vessel formation [30, 31]. Furthermore, the KEGG pathway analysis (Fig. 6f) revealed significant enrichment in pathways such as the cell cycle and PI3K-Akt signaling pathways, which are closely associated with angiogenesis [32, 33].

### miR@TDN-enhanced dental pulp regeneration in vivo

**Fig. 7:**
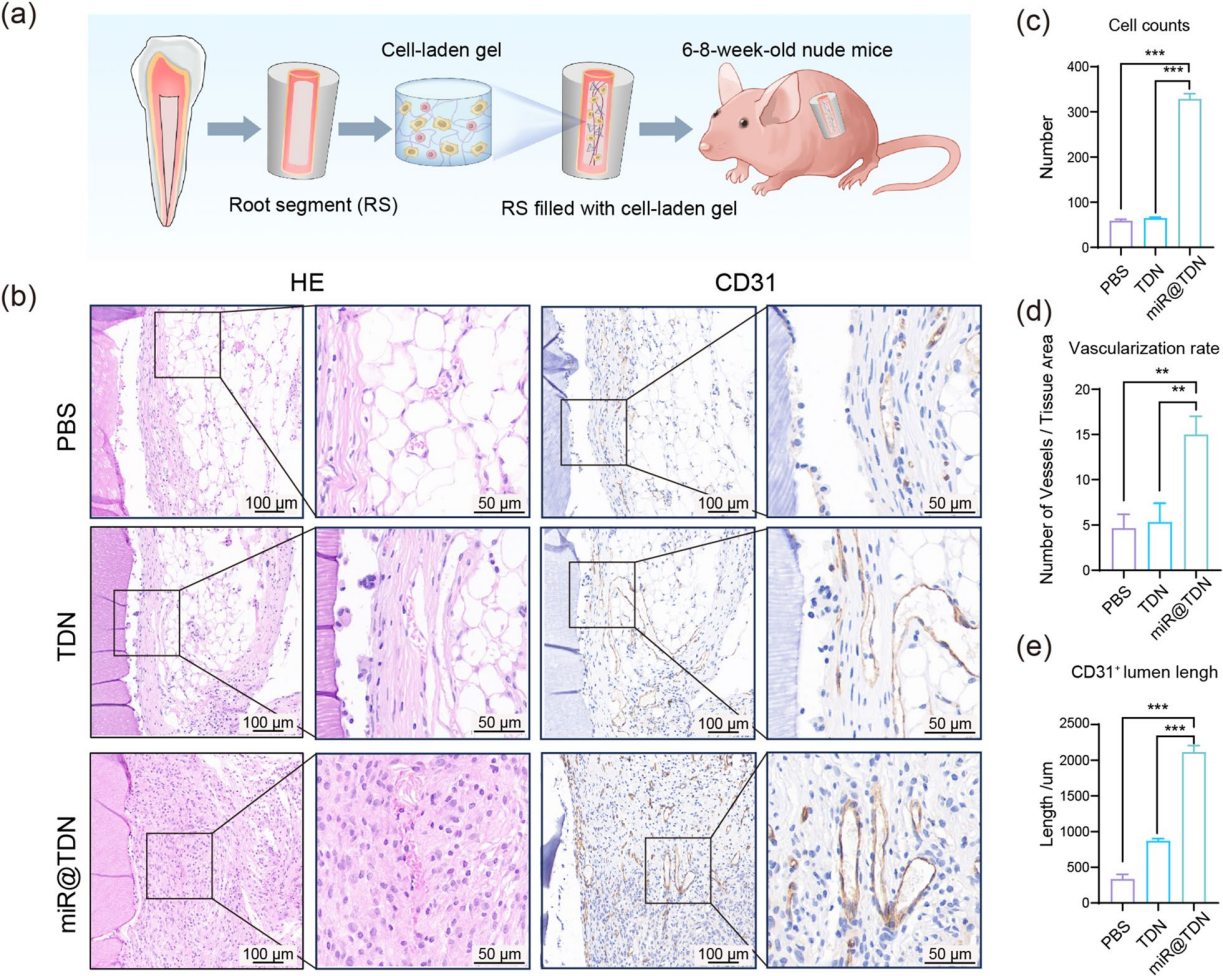
miR@TDN-induced enhanced angiogenesis/vasculogenesis in an in vivo dental pulp regeneration model: **a** The schematic diagram illustrates the ectopic pulp regeneration model in the nude mice. A mixture of collagen gel, DPSCs (pre-treated by miR@TDN) and HUVECs are injected into the inner space of the RSs. Subsequently, these RSs are subcutaneously transplanted into the backs of nude mice and allowed to regenerate for 6 weeks. **b** The representative images depict the retrieved RSs and the HE-stained sections, and the results of IHC staining to visualize human CD31 expression as a marker for blood vessels. **c**, **d**, **e** Results of quantitative assessment, which include the cell counts, vascularization rates (from HE-stained sections in panel **b**) and $$\hbox {CD31}^{+}$$ lumen lengths (from IHC staining in panel **b**). The data are obtained through triplicate experiments ($$\hbox {n}=3$$) and are expressed as the mean ± SD. The statistical analysis shows that the significance levels are denoted as follows: $$**p<0.01, **p <0.001$$, NS indicates no significance

Building on previous research, we conducted a classic dental pulp ectopic transplantation experiment to further evaluate the potential of miR@TDNs in promoting dental pulp regeneration [34]. We pretreated DPSCs using distinct conditions for each group: the PBS, TDN, and miR@TDN groups were treated with PBS, TDN (200 nM), and miR@TDN (200 nM), respectively. Subsequently, the pretreated DPSCs were co-cultured with HUVECs at a 3:1 ratio, reflecting the designated cell proportions [35]. The resulting DPSC–HUVEC composite was embedded in a Type I collagen hydrogel (medgel), forming a cell-laden gel structure. The composite was then injected into the inner space of pretreated root segments (RSs), followed by a 48-h in vitro incubation and subsequent subcutaneous implantation into nude mice (Fig. 7a). This implantation setup provided a controlled setting for observing the subsequent dental pulp regeneration process, allowing for a 6-week regenerative period. The histological analysis of the H &E-stained sections revealed marked disparities in the tissue morphology and organization across the experimental groups. Notably, the miR@TDN group showed the most favorable regenerative outcomes, characterized by well-organized cellular clusters and extensive extracellular matrix deposition (Fig. 7b). The tissue architecture displayed a compact and densely populated pattern, suggesting increased cellular proliferation and structural alignment. Conversely, the TDN group exhibited intermediate regenerative features, including noticeable cellular clustering and moderate extracellular matrix deposition. Although the tissue architecture appeared relatively organized, it was less structured than that in the miR@TDN group. In contrast, the vehicle group demonstrated a less pronounced regenerative response with dispersed cellular distribution and limited extracellular matrix deposition. The quantitative analyses of the cell counts and vascularization rates (the number of blood vessels per square millimeter, and it was calculated as the number of vessels divided by tissue area) showed that the miR@TDN group had significantly outperformed the other two groups (Fig. 7c, d) [23]. The results of IHC staining that targeted CD31, a validated marker of endothelial cells and angiogenesis, provided insights into vascularization within the regenerating dental pulp. Strikingly, the miR@TDN group exhibited robust CD31 staining, indicating increased angiogenesis and neovascularization (Fig. 7b). The abundance of $\hbox {CD31}^{+}$ vessels in the miR@TDN group underscored the potent proangiogenic effects of miR@TDN treatment. The quantitative analysis of the $\hbox {CD31}^{+}$ lumen length further confirmed the significant superiority of the miR@TDN group (Fig. 7e). Following transplantation, neighboring host cells could migrate through the apex of the root canal. To confirm that the regeneration of dental pulp tissue was initiated by the implanted DPSCs and HUVECs, we performed immunofluorescence analysis using human mitochondrial antibodies, specifically labeling the mitochondria of the human cells, but not those of the mouse cells. The results revealed the widespread expression of human mitochondrial antigen in the regenerated tissue, especially close to the blood vessels (Additional file 1: Fig. S6). Regarding the developed blood vessels, there were no significant differences in the proportion of vessels expressing human mitochondria between the three groups, suggesting that the regenerated dental pulp tissue had been predominantly formed by the implanted cells.

## Discussion

The regeneration of dental pulp holds immense significance in restoring tooth function and aesthetics [1]. Stem cell-based approaches, particularly using dental pulp stem cells (DPSCs), have shown promise in this regard. DPSCs, strategically located within the dental pulp microenvironment, play a pivotal role in angiogenesis, essential for the rapid establishment of functional vascular networks during dental pulp regeneration [6]. MicroRNAs (miRNAs) have emerged as promising tools for driving stem cell-mediated angiogenesis, but their efficient delivery poses challenges [13].

Efficient miRNA delivery, a critical aspect of harnessing their therapeutic potential, has seen significant advancements through chemical modifications. The inspiration for this chemical modification strategy finds resonance in the field of RNA-targeting therapeutics, notably in the case of Inclisiran, a chemically modified drug. The use of 2’-O-methyl and 2’-Fluoro modifications, as observed in Inclisiran, has proven effective in improving the pharmacokinetic and pharmacodynamic properties of RNA molecules [12]. The success of Inclisiran underscores the transformative potential of chemically modified RNA in therapeutic applications. In the specific context of this study, the exploration of three chemically modified variants of miR-126-3p highlights the ongoing efforts to optimize miRNA therapeutics. The selection of 126-Mod1, based on its superior stability and increased angiogenic potential, draws parallels with successful strategies employed in other miRNA-based applications.

The delivery of miRNAs is a critical aspect of their therapeutic application, and the study introduces a novel delivery system, miR@TDNs (tetrahedral DNA nanostructures), characterized by a unique triangular structure. The successful synthesis of miR@TDNs, characterized by a unique triangular structure, was achieved through the conjugation of miR-126-3p (126-Mod1) with tetrahedral DNA nanostructures (TDNs). The stability of miR@TDNs in different conditions was confirmed, ensuring their viability for in vitro and in vivo applications. Cellular uptake studies demonstrated the superior efficiency of miR@TDNs compared to unlinked miRNAs, emphasizing their potential for enhanced intracellular delivery. In contrast to alternative delivery methods [36, 37], miR@TDNs offer several distinct advantages. Firstly, they ensure safety by demonstrating no observed cellular toxicity, a critical factor for the success of miRNA-based therapies. Moreover, miR@TDNs demonstrate exceptional delivery speed, swiftly entering cells to significantly enhance overall delivery efficiency. This feature underscores their effectiveness in efficiently transporting miRNAs into target cells, a crucial aspect for the success of therapeutic applications.

DPSCs, crucial contributors to angiogenesis, play a vital role in the collaborative effort of various cell types, notably emphasizing the role of epithelial cells, for blood vessel formation [38]. Understanding their paracrine effects is key, recognizing that vascular development involves coordinated actions among multiple cell types. Previous studies on DPSC paracrine effects offer valuable insights into this intricate process [2, 39], highlighting the significance of DPSCs in regulating angiogenesis within the broader context of vascular development. The conditioned medium from miR@TDN-treated DPSCs exhibited a potent angiogenic effect on HUVECs, as demonstrated by tube formation and matrigel plug assays showcasing substantial enhancements in angiogenic indicators. These findings strengthened the notion that miR@TDNs had promoted the formation of functional blood vessels. The matrigel plug assay results provided a comprehensive picture of the role of miR@TDNs in angiogenesis. Although there is currently no definitive evidence suggesting that miR@TDNs directly influence intracellular angiogenesis-related signaling pathways within DPSCs, we propose several hypotheses to explain this promotional effect. First, miR@TDNs may enhance the angiogenic potential of DPSCs by modulating specific cell signaling pathways through the delivery of microRNAs. Evidently, miRNAs such as miR-126-3p play crucial roles in influencing cell functions by targeting key molecules within various signaling pathways, particularly those related to angiogenesis [40, 41]. These miRNAs have been shown to regulate angiogenesis by blocking specific inhibitory factors such as sprouty-related EVH1 domain-containing protein 1 and modulating the expression of angiogenic factors such as VEGFs [42, 43]. Furthermore, the nanotechnology employed in miR@TDNs may enhance the stability and delivery efficiency of miR, thereby strengthening its action within DPSCs. This technology supports the effective intracellular delivery of miRNAs, thereby affecting cell function more positively. Finally, it is noteworthy that the standalone application of the TDN framework itself has been reported to independently promote angiogenesis [44].

Transcriptome analysis uncovered profound changes in gene expression profiles, notably upregulating crucial angiogenic factors and pathways. This molecular shift aligns with the observed enhancement in DPSC proliferation and angiogenesis-related outcomes.Noteworthy genes, including HIF1 $\alpha$, Platelet-Derived Growth Factor D, and angiopoietin 1, were significantly upregulated, indicating that miR@TDN treatment stimulated the production of essential growth factors pivotal for cell proliferation and new blood vessel formation [30, 31]. The broad upregulation of genes associated with cell proliferation, such as cyclins and cyclin-dependent kinases, suggests that miR@TDNs play a role in promoting robust DPSC growth and proliferation [45].Furthermore, KEGG pathway analysis (Fig. 6f) highlighted significant enrichment in pathways like the cell cycle and PI3K-Akt signaling, intricately associated with angiogenesis. The intricate interplay of miR@TDNs with these pathways warrants detailed exploration in future studies, offering prospects for deeper insights into the mechanisms underpinning enhanced dental pulp regeneration.

Histological analysis of the dental pulp ectopic transplantation model, a variation of R.B. Rutherford’s work on dental tissue regeneration developed by Huang’s Laboratory [46], confirmed the regenerative potential of miR@TDNs. This semiorthotopic model, closely mirroring clinical conditions, allows for pulp–dentin regeneration within an actual tooth. Despite the advantages of simplicity and providing an orthotopic-like environment, it presents notable limitations. The variance in blood supply between mouse subcutaneous tissues and periapical tissues, differing operating procedures on tooth samples compared to clinical practices, and the involvement of mouse cells in regenerated tissues are key considerations [47]. Therefore, addressing the long-term stability of regenerated tissue and host interactions, along with exploring large animal in situ transplantation experiments, should be priorities in future investigations.

## Conclusion

In summary, we successfully synthesized novel nanoparticles called miR@TDNs, which were used to promote dental pulp regeneration by activating DPSCs. Our results indicated that miR@TDNs could significantly enhance the proliferation and migration abilities of the DPSCs. They upregulated genes related to angiogenesis in DPSCs, thereby enhancing their paracrine signaling effects and consequently improving the vascular formation abilities of the endothelial cells. The transcriptome analysis results further revealed the upregulation of angiogenesis-related genes and enrichment of pathways closely related to angiogenesis as potential mechanisms by which miR@TDNs could promote dental pulp regeneration. Furthermore, in the context of ectopic dental pulp transplantation, our findings unequivocally demonstrated the remarkable efficacies of miR@TDNs in facilitating angiogenesis and fostering dental pulp regeneration. Overall, the miR@TDNs efficiently delivered chemically modified miR-126-3p, facilitating the behavior and paracrine signaling of DPSCs and ultimately enhancing angiogenesis both in vitro and in vivo. These findings highlighted the potential of miR@TDNs as powerful tools for gene therapy and their prospective use in regenerative dentistry.

### Supplementary Information

**Additional file 1.** The additional information contains supplementary experimental methodologies and details utilized in the study. It covers various procedures such as Cellular Uptake of Nanostructures, Cell Proliferation Assay, miRNA Transfection, RNA Sequencing (RNA-seq), Bioinformatics Analysis, Total RNA isolation, qPCR analysis, and Western Blotting. Additionally, it includes assessments such as evaluating the angiogenic potential of specific microRNAs (let-7a, miR-21-3p, miR-126-3p, and miR-210), examining the uptake of miR@TDN by DPSC, analyzing vascular-related gene expression in DPSCs and HUVECs following different interventions, and displaying immunofluorescence images showing the presence of human mitochondria within blood vessels formed in transplants in Root segments (RSs).

## Data Availability

Not applicable.
