# Supplementary material for: Chemically modified microRNA delivery via DNA tetrahedral frameworks for dental pulp regeneration
Source: J Nanobiotechnology. 2024 Apr 4;22:150. doi: 10.1186/s12951-024-02393-9 (PMC11318316; doi:10.1186/s12951-024-02393-9)
Supplement: Supplementary file 1 — Additional file 1. The additional information contains supplementary experimental methodologies and details utilized in the study. It covers various procedures such as Cellular Uptake of Nanostructures, Cell Proliferation Assay, miRNA Transfection, RNA Sequencing (RNA-seq), Bioinformatics Analysis, Total RNA isolation, qPCR analysis, and Western Blotting. Additionally, it includes assessments such as evaluating the angiogenic potential of specific microRNAs (let-7a, miR-21-3p, miR-126-3p, and miR-210), examining the uptake of miR@TDN by DPSC, analyzing vascular-related gene expression in DPSCs and HUVECs following different interventions, and displaying immunofluorescence images showing the presence of human mitochondria within blood vessels formed in transplants in Root segments (RSs). [file 12951_2024_2393_MOESM1_ESM.pdf]

Support Information

Chemically Modified MicroRNA Delivery via DNA Tetrahedral Frameworks for Dental Pulp Regeneration

Xiaoling Wei[1,2†], Huaxing Xu[1,2†], Mengqi Zhou[1,2], Qiangqiang Zhou[1,2], Mingqiang Li[3*], Yuehua Liu[1,2*]

[1]Shanghai Stomatological Hospital&School of Stomatology, Fudan University, Shanghai, 200001, China.
[2]Shanghai Key Laboratory of Craniomaxillofacial Development and Diseases, Fudan University, Shanghai, 200001, China.
[3]School of Chemistry and Chemical Engineering, New Cornerstone Science Laboratory, Frontiers Science Center for Transformative Molecules, National Center for Translational Medicine, Shanghai Jiao Tong University, Shanghai, 200240, Shanghai.

*Corresponding author(s). E-mail(s): limingqiang@sjtu.edu.cn; liuyuehua@fudan.edu.cn;
†These authors contributed equally to this work.

S1 Supplementary Methodology

S1.1 Cellular Uptake of Nanostructures

The drug was introduced to study the endocytosis of miR@TDNs, and observations were conducted at 30- and 60-min. The miR@TDN concentration used in these experiments was 200 nM. Qualitative assessments were performed by fluorescence staining and laser confocal microscopy (FV3000; Olympus). The quantitative analysis of the fluorescence intensity was conducted using a flow cytometer (FACSCanto, BD). Then, the data were processed and evaluated using the FlowJo X.

S1.2 Cell Proliferation Assay

The effects of miR@TDNs at various concentrations (12.5, 25, 50, 100, and 200 nM) on the DPSCs were assessed. Initially, CCK-8 cell viability assay (CCK-8) was used to evaluate DPSC proliferation. The DPSCs were cultured in αMEM (5% FBS) with the specified miR@TDN concentrations (treatment groups), while 5% αMEM (5% FBS) was used as the control. The optical density was measured at 450 nm at 12, 24, 36, 48, 72, 96, and 120 h using a microplate spectrophotometer (Thermo Fisher Scientific).

S1.3 miRNA Transfection

The cultured DPSCs were harvested and seeded into culture vessels to reach the desired confluency. The miR@TDN and Lipofectamine 3000 were mixed separately with the culture medium. Then, they were combined and incubated to form complexes. The transfection mix was added to DPSCs and incubated. The DPSCs were incubated with the transfection mix to allow the complexes to enter the cells. After incubation, the transfection mix was replaced with fresh growth medium. Subsequently, RNA extraction and quantitative real-time polymerase chain reaction(qPCR) were performed to confirm that the transfection had been successful.

S1.4 RNA Sequencing (RNA-seq) and Bioinformatics Analysis

DPSCs were treated with miR@TDNs (200 nM), with untreated DPSCs serving as controls. Total RNA was extracted using TRIzol® Reagent (Invitrogen) and sequenced on the Illumina NovaSeq 6000 platform at Shanghai Majorbio Bio-Pharm Biotechnology Co., Ltd., per Illumina's guidelines. PE150 sequencing was subjected to quality control via fastp. The alignment of reads to the genome was performed using HISAT2, and DEGs were identified by StringTie. Expression levels were quantified using TPM and RNA-Seq by Expectation-Maximization. DEGs were detected using DESeq2 and DEGseq ($|\log_2 \mathrm{FC}| \geq 1$, $FDR < 0.05$ or $FDR < 0.001$). Functional enrichment analysis, including GO and KEGG), was performed using a Bonferroni-corrected p-value ¡ 0.05, implemented via GOATOOLS and Python SciPy. Reactome enrichment analysis was conducted using Python software.

S1.5 Total RNA isolation and qPCR analysis

Total cellular RNA was extracted using QIAzol and purified using an RNeasy Mini Kit (QIAGEN). Total RNA (2 μg) was reverse transcribed using an iScript cDNA synthesis kit (Bio-Rad). Subsequently, qPCR analysis was conducted using SYBR Green qPCR Master Mix (Yeaso) and a real-time PCR system (Roche). The primer sequences are listed in Supplementary Table S2. For miR-126-3p detection, the reverse transcription (RT) was performed using a Stem-loop primer method. This method uses a reverse transcription primer with a Stem-loop structure (5'-GTCGTATCCAGTGCAGGGTCCGAGGTATTCGCACTGGATACGAC TTTTTTTT UCGUACCGUGAGUAAUAAUGCG-3') that can specifically bind to the target miRNA, enabling its detection with high specificity and sensitivity.

S1.6 Western Blotting

Western blot (WB) analysis was conducted according to established protocols. Briefly, tissues and cells were homogenized, separated by sodium dodecyl sulfate-polyacrylamide gel electrophoresis, and transferred onto a polyvinylidene fluoride membrane. After being incubated in a blocking buffer with 5% of skim milk for 1 h at 37 °C, the membrane was exposed to specific primary monoclonal antibodies, including endothelial nitric oxide synthase (eNOS) (1:1000; Proteintech), cluster of differentiation 31 (CD31) (1:3000; Proteintech), vascular endothelial growth factor A (VEGFA) (1:3000; Proteintech) and hypoxia-inducible factor 1-alpha (HIF1α) (1:200, Santa Cruz overnight at 4 °C). Relative secondary antibodies (1:2000; Beyotime) were incubated with primary antibodies for 1 h at 37 °C. After three washes with Tris-buffered saline containing tween (TBST), each band was visualized using an enhanced chemiluminescence (ELS) detection system (Bio-Rad). Glyceraldehyde 3-phosphate dehydrogenase served as an internal control.

S2 Supplementary Tables and Figures

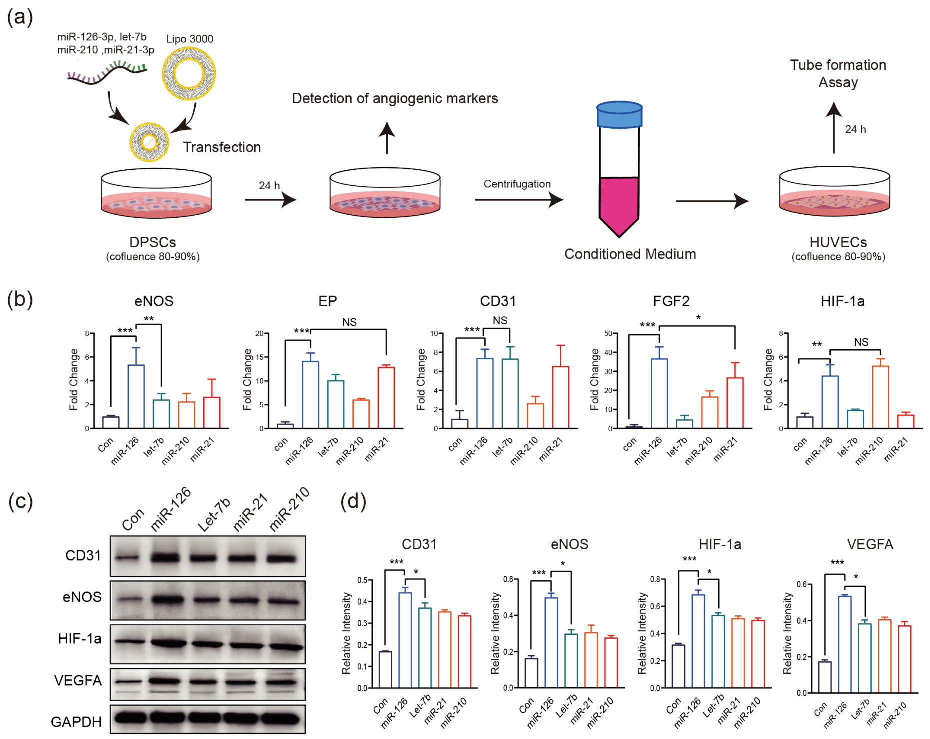

Figure S1: Screening of angiogenesis-promoting microRNAs. (a) Schematic representation of the microRNA screening process. (b) The expression levels of angiogenesis-related genes were measured using quantitative polymerase chain reaction (qPCR) following transfection of let-7a, miR-21-3p, miR-126-3p, and miR-210 into DPSCs (Dental Pulp Stem Cells). (c) the effects of let-7a, miR-21-3p, miR-126-3p, and miR-210 transfection on the expression of angiogenesis-related proteins were evaluated using Western blotting (WB) analysis. (d) The presentation of related proteins in (c) was quantitatively analyzed. Data includes the mean ± SD, statistical analysis: $*p < 0.05, **p < 0.01, ***P < 0.001$, NS, no significance.

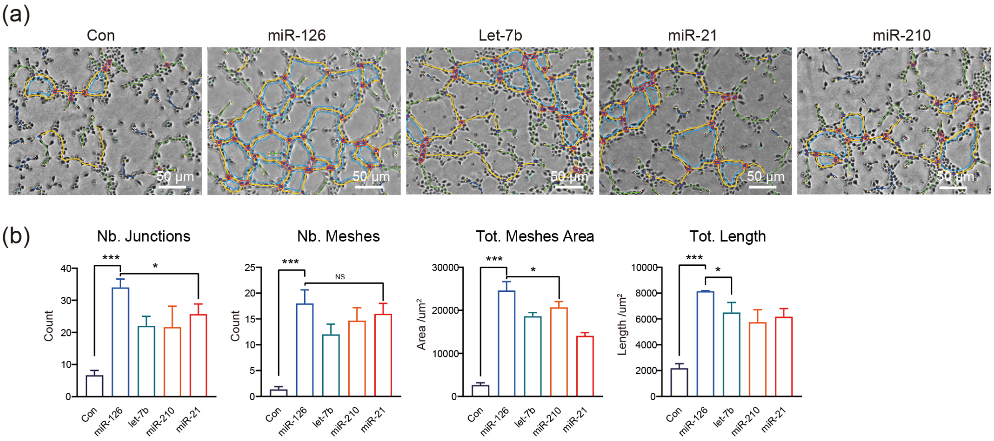

Figure S2: Evaluation of the angiogenic potential of the four microRNAs (let-7a, miR-21-3p, miR-126-3p, and miR-210). (a) tube formation assay. (b) quantitative analysis of the tube formation assay images. Data includes the mean ± SD, statistical analysis: $*p < 0.05, ***P < 0.001, NS, nosignificance.$

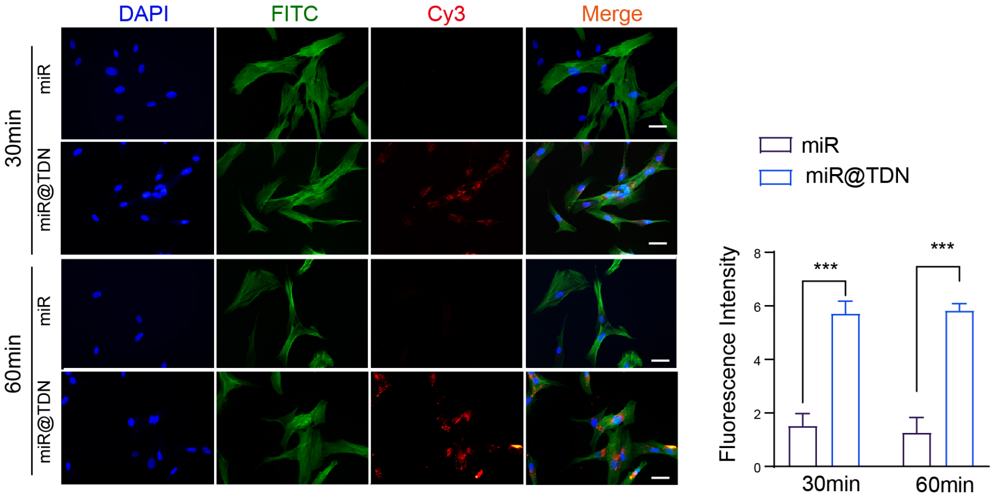

Figure S3: Fluorescence analysis demonstrates miR@TDN uptake by DPSCs (Red: Cy3-miR or Cy3-miR@TDN, Blue: DAPI-nuclei; Green: FITC-cytoskeleton. Scale bars: 20μm), presenting quantitative statistics of fluorescence intensity (n=3). Data comprise mean values with ± SD, statistical analysis: $***p < 0.001$.

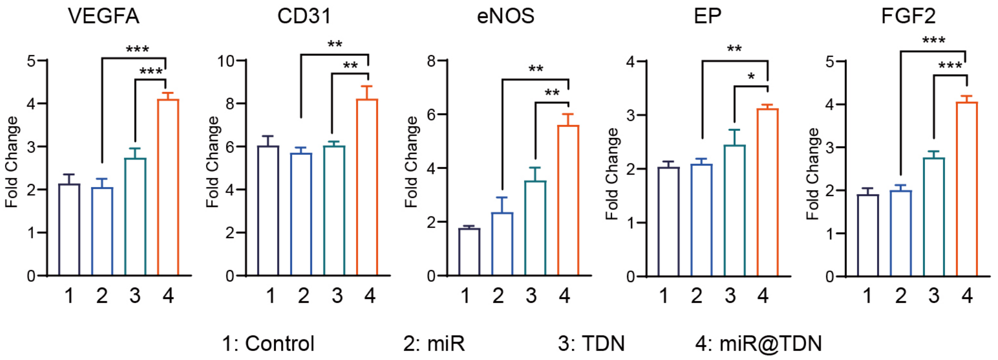

Figure S4: Evaluation of vascular-related genes expression in DPSCs following miR, TDN, and miR@TDN interventions by qPCR, n=3. Data include the mean ± SD, statistical analysis: $*p < 0.05, **p < 0.01, ***p < 0.001$.

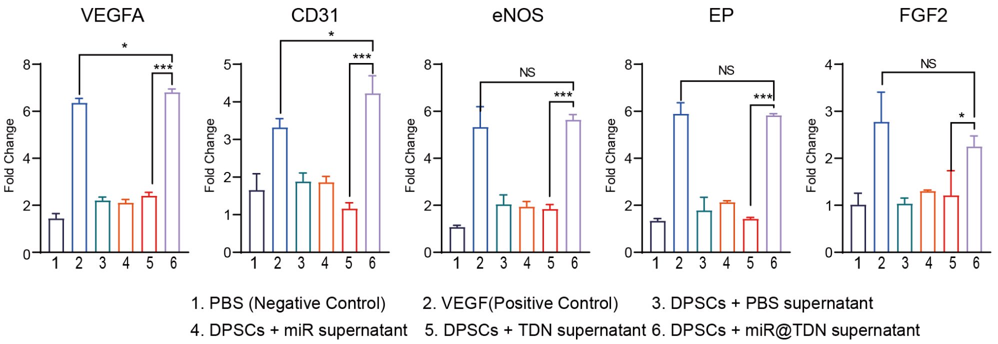

Figure S5: Results of qPCR analyses of the expression of vascular-related genes in HUVECs following six different interventions, n=3.Data include the mean ± SD, statistical analysis: $*p < 0.05, ***p < 0.001$, and NS indicates no significance.

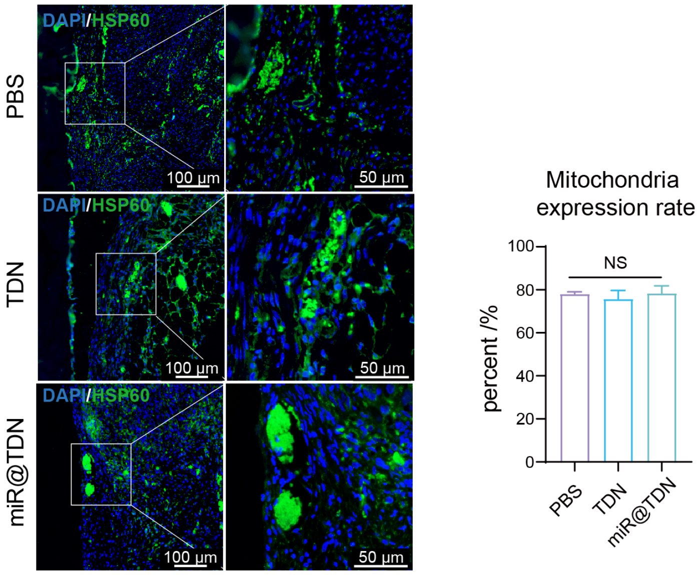

Figure S6: Immunofluorescence images illustrate the presence of human mitochondria (green) within transplants' blood vessels, highlighting the contribution of transplanted cells to vasculature formation. Quantitative assessment shows the proportion of regenerated blood vessels expressing human mitochondria. Data, acquired from triplicate experiments (n=3), are presented as mean ± SD. The statistical analysis shows that the significance levels are denoted as follows: NS indicates no significance.

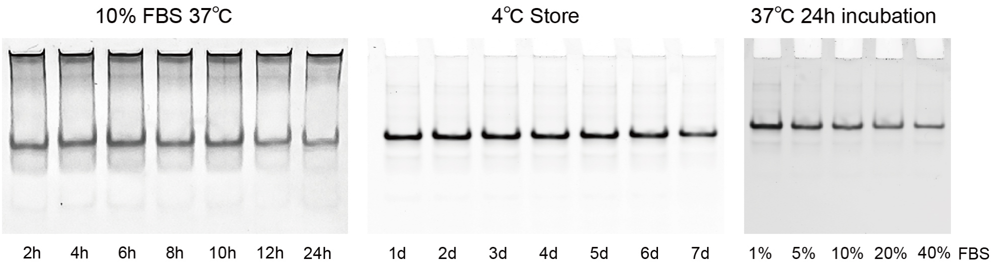

Figure S7: The full gel image for Figure 2e. Structure stability of miR@TDN (left: Serum stability of miR@TDNs within 24 h in 10 % FBS; Middle: Storage stability of miR@TDN at 4°C; Right: Stability of miR@TDNs in different concentrations of FBS).

Table S1: Sequences of the designed ssDNAs and miR-126-3p

ssDNA	Sequence (5'-3')
T1	ATTTATCACCCGCCATAGTAGACGTATCACCAGGCAGTTGAGACGAACAT TCCTAAGTCTGAA
T2	ACATGCGAGGGTCCAATACCGACGATTACAGCTTGCTACACGATTCAGAC TTAGGAATGTTCG
T3	ACTACTATGGCGGGTGATAAAACGTGTAGCAGCTGTAATCGACGGGAAG AGCATGCCCATCC
T4	ACGGTATTGGACCCTCGCATGACTCAACTGCCTGGTGATACGAGGATGG GCATGCTCTTCCCG
T4COM	ACGGTATTGGACCCTCGCATGACTCAACTGCCTGGTGATACGAGGATGG GCATGCTCTTCCCGAGCATGGCACTCATTATTACGC
miR-126-3p	UCGUACCGUGAGUAAUAAUGCG

Table S2: Primers of target genes

Primer	Primer pairs (5'-3')
GAPDH	Forward: GGTGAAGGTCGGTGTGAACG<br>Reverse: CTCGCTCCTGGAAGATGGTG
VEGFA	Forward: CTGCTGTGGACTTGTGTTGG<br>Reverse: AAAGGACTTCGGCCTCTCTC
CD31	Forward: AAGTGGAGTCCAGCCGCATATC<br>Reverse: ATGGAGCAGGACAGGTTCAGTC
eNOS	Forward: GAAGGCGACAATCCTGTATGGC<br>Reverse: TGTTCGAGGGACACCACGTCAT
EP	Forward: GCATGTGGATAAAGCCGTCAGTG<br>Reverse: GAGTTTGCGGAAAGTGTCAGCAG
FGF2	Forward: AGCGGCTGTACTGCAAAAACGG<br>Reverse: CCTTTGATAGACACAACTCCTCTC
